# mTORC1-selective inhibitors rescue cellular phenotypes in TSC iPSC-derived neurons

**DOI:** 10.3389/fnins.2025.1595880

**Published:** 2025-07-28

**Authors:** Elizabeth D. Buttermore, Gayathri Rajaram Srinivasan, Hellen Jumo, Amanda C. Swanson, Benjamin O’Kelly, Nina R. Makhortova, Mustafa Sahin, Stelios T. Tzannis

**Affiliations:** ^1^Human Neuron Core, Rosamund Stone Zander Translational Neuroscience Center, Boston Children’s Hospital, Boston, MA, United States; ^2^F.M. Kirby Neurobiology Center, Boston Children’s Hospital, Boston, MA, United States; ^3^Department of Neurology, Harvard Medical School, Boston, MA, United States; ^4^Aeovian Pharmaceuticals, Inc., Berkeley, CA, United States

**Keywords:** mTORC1, mTORC2, iPSC-derived neurons, *TSC2*, mTOR, hyperexcitability, soma size

## Abstract

The mechanistic target of rapamycin (mTOR) pathway plays an important role in regulating multiple cellular processes, including cell growth, autophagy, proliferation, protein synthesis, and lipid synthesis, among others. Given the central role of this pathway in multiple cellular processes, it is not surprising that mTOR pathway dysregulation is a key mechanism underlying several neurological disorders, including Tuberous Sclerosis Complex (TSC). TSC patients typically present with pathogenic variants in the *TSC1* or *TSC2* genes, which encode proteins forming a complex that plays an important role in modulating mTOR activity. We previously reported cellular and functional deficits in induced pluripotent stem cell (iPSC)-derived neurons from TSC patients. These deficits were reversed by inhibiting mTOR activity using rapamycin treatment, revealing the role of mTOR signaling in the regulation of cell morphology and hyperexcitability phenotypes in TSC patient-derived neurons. However, chronic rapamycin treatment inhibits both mTORC1 and mTORC2 activity and its clinical use is associated with significant side effects. With the development of novel mTORC1-selective compounds, we aimed to assess whether selective inhibition of mTORC1 likewise reversed the cellular and functional deficits found in TSC patient-derived neurons. Our results indicate that the novel, selective mTORC1 inhibitors nearly fully reversed the cellular and functional deficits of *TSC2*^–/^*^–^* iPSC-derived neurons in a fashion and magnitude similar to rapamycin, as they all reversed and near-normalized their neuronal hyperexcitability and abnormal morphology as compared to the DMSO-treated cells. These data suggest that mTORC1-specific compounds could provide clinical therapeutic benefit similar to rapamycin without the same side effects.

## 1 Introduction

Many neurodevelopmental disorders (NDDs) are associated with changes in the mechanistic target of rapamycin (mTOR) pathway (Parenti et al., 2020). The mTOR pathway regulates proliferation, cell growth, protein synthesis, lipid synthesis, and autophagy, which lead to its important roles in nervous system development, including regulation of progenitor proliferation, neurite outgrowth, and neuronal maturation (Lipton and Sahin, 2014). In addition, the mTOR pathway has been shown to play an important role in synaptic function (Weston et al., 2014). Further, there are well-defined monogenetic variants in genes that code for mTOR pathway proteins that lead to disrupted mTOR signaling and that have been associated with intellectual disability, epilepsy, and autism spectrum disorder (Moloney et al., 2021; Parenti et al., 2020; Reijnders et al., 2017; Vasic et al., 2021; Winden et al., 2018). One example of this is Tuberous Sclerosis Complex (TSC), which is caused by variants in *TSC1* or *TSC2* genes (Rosset et al., 2017). TSC1 and TSC2 proteins form a complex that helps regulate mTOR activity by regulating RHEB, which serves as a brake on the activity of mTORC1, one of the key complexes of mTOR signaling (Huang and Manning, 2008). Researchers have also shown that the TSC1/TSC2 complex affects the activity of a second mTOR-containing complex called mTORC2 (Huang et al., 2008; Huang and Manning, 2009). More recently, it has been shown that reduction of the mTORC1 component Raptor, but not the mTORC2 component Rictor, rebalanced mTOR signaling in *Tsc1* mouse knock-out neurons (Karalis et al., 2022). Therefore, dysfunctional TSC1 or TSC2 disrupts normal regulation of mTORC1 and mTORC2, with several studies pointing toward mTORC1 hyperactivity as a potential driver for disease phenotypes (Costa et al., 2016; Switon et al., 2017).

We have previously utilized TSC patient induced pluripotent stem cell (iPSC)-derived neurons to show that dose-dependent reduction in *TSC2* expression leads to dosage-sensitive cellular changes in neuronal morphology, including changes in gene expression, increased soma size and neurite outgrowth, as well as neuronal hyperexcitability (Winden et al., 2023; Winden et al., 2019). Further, we previously showed that chronic rapamycin treatment could rescue the hyperexcitability phenotype in TSC2-deficient iPSC-derived neurons. While rapamycin, an allosteric mTOR inhibitor, primarily inhibits mTORC1, prolonged treatment with rapamycin or its analogs (termed rapalogs) has been shown to inhibit mTORC2 as well (Lamming, 2016; Sarbassov et al., 2006). Chronic administration of rapalogs has been suggested to cause undesirable side effects such as reduced insulin sensitivity and hyperlipidemia (Bissler et al., 2017; Trelinska et al., 2015). Since mTORC2 influences glucose metabolism and insulin signaling, a subset of these undesirable side effects of long-term rapamycin treatment have been suggested to be mediated due to the inhibition of mTORC2 (Lamming et al., 2012). Therefore, more precise targeting of mTORC1 hyperactivation is desirable for therapeutic development.

Several mTORC1-selective inhibitors have been developed recently (Balgi et al., 2009; Kang et al., 2019; Mahoney et al., 2018; Schreiber et al., 2019) and have been evaluated in a number of immortalized cell lines in the context of tumor reduction or in rodent models *in vivo* (Schreiber et al., 2019) and *in vitro* (Di Nardo et al., 2020) to assess the effects of mTORC1-inhibition in the brain and in neurons, respectively. However, studies describing their effects on the morphology and function of human neurons have not been reported to our knowledge. Human iPSC-based models offer a promising approach to model TSC as well as to provide a relevant platform to study the effects of mTORC1-selective inhibitors (Afshar Saber and Sahin, 2020; Yang et al., 2024). Here, we used *TSC2^–/–^* iPSC-derived neurons to test whether novel mTORC1-selective inhibitors rescued established cellular deficits as effectively as rapamycin. There is a significant volume of published literature studies, both *in vitro* (in PC3 or other cell systems) and *in vivo*, confirming the high mTORC1 selectivity of these novel rapamycin analogs, including the tool compound, DL001 (Schreiber et al., 2019). Here, we also present AlphaLISA data demonstrating this selectivity. When we assessed these compounds in our iPSC-derived neuron model, we found that all three novel compounds rescued the hyperexcitability and neuron morphology phenotypes with similar potency as rapamycin. This suggests that mTORC1 inhibition is sufficient to rescue cellular phenotypes found in *TSC2^–/–^* iPSC-derived neurons.

## 2 Materials and methods

### 2.1 Novel mTORC1-selective compounds

The general Markush structures of the compounds used in this work can be found in the issued US patent US11230557B2. More specifically, the three compounds used in this study differ in the types of substitutions made in the R1, R2, and R3 positions of the broad Markush structure (Supplementary Figure 1). The purity of all compounds produced was greater than 95% for all assays. The general process utilized in the purification of the produced compounds can be found in the examples provided within the issued US patent US11230557B2.

### 2.2 AlphaLISA mTORC1 vs mTORC2 selectivity assay

PC3 cells were cultured in Ham’s F12K medium (Gibco, #21127-022) containing 10% of FBS (Sigma #F7524, batch BCBV4855). PC3 cells were seeded at 20 000 cells/well in 90 μl of culture medium in 96-well plates. 24 h after seeding, cells were treated with compounds for 8 h (1% final DMSO concentration). Cells were washed with PBS and treated for 16 h in starvation medium (Ham’s F12K without FBS). Cells were then stimulated by adding FBS (12% final concentration) and incubated for 15 min. Wells were washed with PBS and 50 μl of AlphaLISA lysis buffer (Revvity) was added. After 30 min of lysis at RT under shaking, 10 μl (p70S6 assay) or 5 μl (pAKT and total AKT assays) of lysate was transferred in 384-well plates. Alpha Lisa assays were performed according to the manufacturer’s instructions (Revvity AlphaLISA Surefire ULTRA P70 S6K #ALSU-PP70-A500, AKT1/2/3 (pS473) #ALSU-PAKT-B500, and ULTRA AKT 1 #ALSU-TAKT1-B500 kits). Briefly, 5 μl of acceptor beads mix was added to each well and incubated for 2 h at RT. Then, 5 μl of donor beads mix was added per well and incubated for 2 h at RT in the dark. Plates were measured on an Envision reader with the AlphaLISA module.

For the AlphaLISA data analysis, the software used was GraphPad Prism version 10.4.1. The equation used is the 4-parameter logistic fit. For the mTORC1 IC50 determination, no constraints were applied in the calculations. For the mTORC2 calculation, the top value was constrained to 100. The equation in GraphPad Prism is denoted as Y = Bottom + (Top - Bottom)/(1 + 10^[[LogEC50 – sX]*HillSlope]).

### 2.3 iPSC lines

The subject recruited for this study was consented at Boston Children’s Hospital with the approved institutional review board protocol number P00008224. Informed consent was obtained from the participant or the participant’s parent if the participant was unable to provide consent. The iPSC line used in this study was previously characterized, including descriptions of reprogramming methods and quality control characterization (Ebrahimi-Fakhari et al., 2016; Sundberg et al., 2018; Winden et al., 2019). Briefly, the *TSC2^–/–^* iPSC line used in this study was originally derived from a female patient with TSC who was found to have heterozygous expression levels of TSC2. The iPSCs were derived by episomal expression of Oct4, Sox2, Klf4, and L-Myc. The second allele was knocked down using TALEN-based gene editing of the *TSC2*^±^ line to generate the biallelic *TSC2^–/–^* line.

The *TSC2^–/–^* iPSCs were previously reported to express the pluripotency markers NANOG, TRA1-60, OCT4, and SSEA4 and exhibit a normal karyotype (Sundberg et al., 2018). For routine cell culture, the iPSCs were cultured in StemFlex medium (ThermoFisher #A3349401) on Geltrex-coated plates (ThermoFisher #A1413301) and were passaged once a week, or when the cells reached 70% confluence, with Gentle Cell Dissociation Reagent (STEMCELL Technologies #07174). A low-passage stock was expanded and karyotyped and all neuronal cultures were derived from iPSCs within 10 passages of the normal karyotype.

### 2.4 Neuronal differentiation

*TSC2^–/–^* iPSC-derived cortical neurons were differentiated in two to three replicate batches, defined as independent plating of starting material iPSCs and use of reagents to differentiate iPSCs into neurons. The NGN2-induced cortical neurons were differentiated following previously published methods (Winden et al., 2019; Zhang et al., 2013) that are also described here. To establish a line of iPSCs expressing the NGN2 vector, on differentiation day - 2, iPSCs were dissociated with Accutase (Innovative Cell Technologies #AT104) and plated as single cells at 90,000 cells/cm^2^ in mTeSR-1 media supplemented with 10 μM ROCK inhibitor (Y-27631, Cayman Chemical #10005583), on Geltrex-coated 12-well plates. While iPSCs were routinely cultured with StemFlex (as noted above), the NGN2 differentiation was optimized with mTeSR-1 media, so the switch was made to this media prior to initiating differentiation. One day after re-plating as single cells, on differentiation day - 1, iPSCs were transduced with lentiviral vectors expressing NGN2 and rtTA and 8 μg/ml polybrene (Sigma-Aldrich #TR-1003-G). The lentiviral particles were produced and concentrated at the Viral Core at Boston Children’s Hospital and were added to the culture at an MOI of 5 for each vector. Both plasmids are available at Addgene with the following IDs: 52047 and 20342. To initiate neuronal induction on differentiation day 0, NGN2 expression was induced via treatment with 2 μg/ml doxycycline (Millipore #324385). Cells then underwent selection for expression of the NGN2 vector via treatment with 1 μg/ml puromycin (Invitrogen #ant-pr-1) for 24–48 h. Longer puromycin treatment was chosen when non-neuronal clusters appeared to remain after the first 24 h of puromycin treatment. This selection helps to generate a relatively pure population of neurons. For the first 2 days of differentiation, the following growth factors and supplements were added to the N2 medium (DMEM/F-12 with GlutaMax, 1x N2 supplement, 1x non-essential amino acids): 10 ng/ml BDNF (Peprotech #450-02), 10 ng/ml NT3 (Peprotech #450-03), and 0.2 mg/L laminin (ThermoFisher #23017-015). After day 2, the cells were fed with B27 media (Neurobasal-A, 1x B27 supplement, 1x GlutaMax) containing the following growth factors and supplements every other day until differentiation day 6: 10 ng/ml BDNF, 10 ng/ml NT3, 0.2 mg/mL laminin, 2 μg/ml doxycycline, and 2 μM Ara-C (Sigma-Aldrich #C1768). For morphological and multi-electrode array (MEA) analyses, NGN2 neurons were dissociated on day 6 with ≥ 16 units/mL papain (Worthington #LK003178) supplemented with ≥ 166 Kunits/mL DNase I (Worthington #LK003172) for 20 min at 37 °C. On day 6, astrocytes (NCardia Astro.4U) were added in co-culture with the neurons. Cells were cultured in B27 media containing 33.4 mM glucose and 27.3 mM sodium bicarbonate, supplemented with 0.1 mg/mL transferrin thereafter with fresh BDNF, NT3 and laminin added at final concentrations listed above, every time media change was performed. Plating formats and conditions were dependent on downstream endpoint and are described in the subsequent methods sections.

### 2.5 Plating for high-content imaging analysis

For high-content imaging assays, neurons were dissociated on differentiation day 6 as described above and were plated along with astrocytes onto 96-well plates (VWR #82050-748) that were first coated overnight with poly-D-lysine (100 μg/mL, Sigma-Aldrich #P6407) followed by three washes with sterile diH_2_O the next day and then coated overnight with laminin (5 μg/mL, Life Technologies #23017-015). Laminin was removed just prior to plating and 50 μL media was added to each well. Neurons were counted using trypan blue stain mixed 1:1 with a small aliquot of cells on a manual hemocytometer, and 20,000 neurons and 3,000 astrocytes were added to each well in a volume of 50 μl per well to reach a final volume of 100 μl per well. Media was supplemented with Rock Inhibitor (Y-27632 #10005583, Cayman) for the first 24 h of plating. The inner 60 wells of each plate were used. Following 3 weeks of maturation and treatment, with half media changes every Monday/Wednesday/Friday, the 96-well plates were fixed with 4% paraformaldehyde (#15686, Electron Microscopy Sci.) for 20 min at room temperature, 1 day after completion of chronic drug treatment.

### 2.6 Plating for multi-electrode array (MEA) analysis

On day 6 of the differentiation protocol for each replicate batch, the neurons were plated onto three full 48-well CytoView MEA plates (M768-tMEA-48B, Axion Biosystems). These plates were coated with 0.1% PEI (#408727, Sigma) and 5 μg/mL laminin. Cortical neurons were plated at 75,000 neurons and 11,250 astrocytes (NCardia, Astro.4U) per well. To plate the neurons, laminin was aspirated off the wells one well at a time and the neuron and glia mixture was added to the well in a 20 μl droplet. Following 30 min of incubation at room temperature, the wells were flooded with 400 μL of media and moved to the incubator. The media at the time of re-plating was supplemented with Rock Inhibitor (Y-27632 #10005583, Cayman) and laminin (10 μg/mL) to promote attachment. A half media change was completed 24 h after plating to dilute the rock inhibitor and half media changes continued every Monday/Wednesday/Friday thereafter.

### 2.7 Compound treatment

**FIGURE 1:**
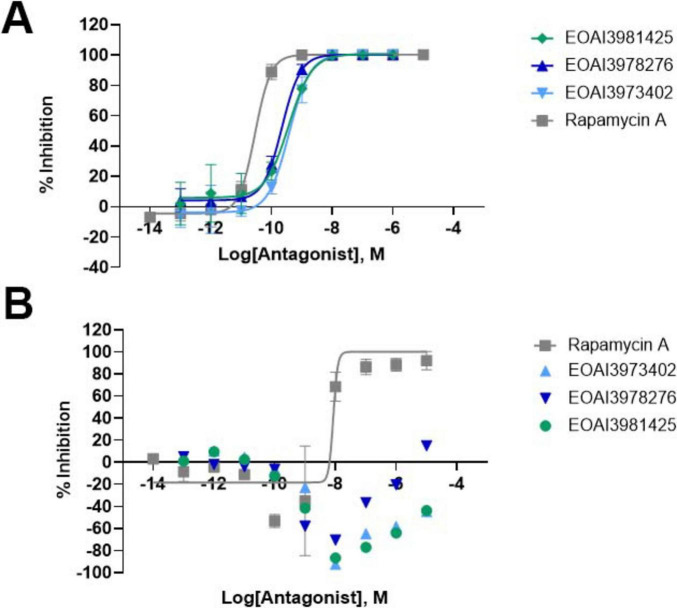
AlphaLISA mammalian target of rapamycin complex 1 (mTORC1) and mammalian target of rapamycin complex 2 (mTORC2) assays reveal mTORC1-selectivity of novel compounds. **(A)** AlphaLISA data showing percent inhibition of P70 S6K phosphorylation as a readout of mTORC1 activity using PC3 cell lysates collected following compound treatment in dose-response. **(B)** AlphaLISA data showing percent inhibition of AKT1/2/3 (pS473) phosphorylation as a readout of mTORC2 activity using PC3 cell lysates collected following compound treatment in dose-response.

Cells were treated with varying concentrations of rapamycin or the mTORC1-selective inhibitor compounds starting from 0.01 nM up to 300 nM, every 2–3 days during the 2 weeks treatment window as indicated in Figure 1A. DMSO treatment was used as control in each plate. We sought to establish a 6-point and a 10-point dose-response curve for the mTORC1-selective inhibitors in MEA experiments and high-content imaging assays respectively. Rapamycin treatments were carried out at 11 different concentrations. A higher number of doses was chosen for rapamycin treatment to gauge the appropriate range of treatment concentrations for the novel mTORC1-selective inhibitors.

Stock vials of rapamycin and mTORC1-selective inhibitors were stored at −20°C, in the dark and were equilibrated to room temperature prior to use. The compounds were reconstituted in DMSO to prepare a 5 mM stock. The 5 mM stock was further diluted to 0.5 mM and 5 μM in DMSO. Each of these stocks were then used to prepare an intermediate stock in DMSO, 1,040 times as concentrated as the required final concentrations ranging from 0.01 to 300 nM. These 1,040× intermediate stocks were then diluted 520-fold in culture media to result in a 2× stock that the cells were finally treated with. All compounds were vortexed at each of the steps described above to ensure full reconstitution/even mixing prior to dilution.

Before addition of the compounds, the media in the 48-well MEA and in 96 well plates for high-content imaging were brought to half the final volume; from 400 to 200 μl for the MEA plates and from 200 to 100 μl for the 96-well plates. To ensure equal volumes of media in all wells across a plate and to account for increased media evaporation from wells on the edge of the plates, a reference well was chosen at random for each plate and most media from this well was removed, leaving behind only a thin film of media in the well. In 48-well MEA plates, 150 μl of the media was carefully added back to this reference well and for 96-well plates, 90 μl of the media was added back so that the media volume in the well would be close to 200 and 100 μl respectively. The media levels in all the other wells of the plate were then visually compared against this reference well to ensure uniformity. The 2× compound stocks prepared in culture media as described above were then carefully added to bring the final volume to 400 μl in MEA plates and 200 μl in 96-well plates, thereby, ensuring incubation with the desired concentrations of the compounds. A different reference well was chosen at each time point to avoid bias.

### 2.8 MEA recording and analysis

Multi-electrode array recordings were performed on an Axion Maestro Original system every 2–3 days, starting day 2 after plating until day 42, according to the protocol and settings detailed in Winden et al. (2019). Briefly, the MEA plates were placed in the recording chamber at 37°C/5% CO_2_ and were allowed to equilibrate for 10 min prior to recording spontaneous activity for 5 min. The Neural software module in Maestro system was used to assess spontaneous activity. A sampling frequency of 12.5 kHz and a 200 Hz high-pass and 3,000 Hz low-pass filter were used to acquire/filter the data. The threshold for spike detection was six times the standard deviation with a 1 s binning time. Network bursts were detected with the following settings: a maximum interspike interval of 100 ms, with a minimum of 50 spikes detected in at least 35% electrodes. Synchrony was detected with a 20 ms window size. After recording, the data across all time points were batch processed, and the weighted mean firing rate and synchrony index were analyzed and exported using Axion Neurostatistics compiler and plotted using GraphPad Prism. Outliers from erroneous recordings were excluded wherever indicated in the figure legends. The fit curves for the neuronal MEA data were constructed using GraphPad Prism software version 9.4.1. The data for a particular compound was entered into an XY graph with X values containing the concentrations of the compound and the Y values including eight technical replicates per compound per time point. The equation used was the three-parameter logistic fit. No constraints were applied in the calculations. The equation in GraphPad Prism is denoted as Y = Bottom + (Top–Bottom)/(1 + [X/IC50]).

### 2.9 Immunocytochemistry

Immunostaining was completed by first blocking neurons with a blocking buffer containing 5% normal goat serum (#G9023-10ML, Sigma), 2% bovine serum albumin (#AB00440-00100, Fisher Scientific), and 0.1% Triton-X (#AC215682500, Fisher) in 1XPBS for one hour. Cells were incubated with primary antibodies in blocking buffer at 4°C overnight. The primary antibodies used are listed in Table 1. Following primary antibody incubation, cells were washed three times with 1XPBS and incubated with fluorophore-conjugated secondary antibodies (Invitrogen) for 1 h at room temperature, followed by three washes. Secondary antibodies used include goat anti-mouse Alexa Fluor 568 (Invitrogen by ThermoFisher #A11031), goat anti-chicken Alexa Fluor 488 (Invitrogen by ThermoFisher #A11039), and goat anti-mouse Alexa Fluor 488 (Invitrogen by ThermoFisher #A11029). In addition to the secondary antibodies, Hoechst 33258 (Invitrogen #H3569) was also added to counterstain the nuclei. Following staining, plates were stored at 4 °C in 1XPBS with 0.01% sodium azide (#S2002, Sigma). Neurons were imaged on the ImageXpress Micro-Confocal automated microscope (Molecular Devices).

**TABLE 1:** Antibody information for high content imaging.

Antibody target	Antibody species	Catalog #	Dilution to use
MAP2	Chicken	Abcam #ab5392	1:2,000
Tuj1	Mouse	Sigma #T6880	1:800

### 2.10 Automated microscopy

All high content imaging was completed using the 10X objective on the ImageXpress Micro-confocal automated microscope from Molecular Devices. The focus settings were established according to the specifications of the plate used. The exposure settings were established on the DMSO-treated wells and the same exposure was utilized across all wells with the same antibody combination. For each treatment condition, 10 wells were imaged in each batch, with two wells per dose and with nine fields captured per well. Following imaging, analysis was completed using the MetaXpress software.

Brightness and contrast for representative images were adjusted using ImageJ and Adobe Photoshop as follows: since one 96 well plate of neurons was used for each compound, to account for variability across plates, the brightness and contrast of the images from the DMSO-treated wells from all the plates were adjusted on ImageJ such that they were visually similar to each other. Next, the brightness and contrast settings applied to the DMSO-treated wells of each plate were applied to all the wells from that specific plate. Representative images from different plates were, thus, included in the figures. The brightness/contrast were further adjusted for these representative images on Photoshop with the same settings applied to each of the channels in a figure panel.

### 2.11 Image analysis

Neuron morphology analysis was completed using the Neuronal Profiler Application Module in the MetaXpress software. This application provides quantification of neuron cell count, soma size, and neurite length using MAP2 staining. The fit curves for the neuronal high content imaging data were constructed using GraphPad Prism software version 9.4.1. The data for a particular compound was entered into an XY graph with X values containing the concentrations of the compound and the Y values including 18 technical replicates per compound per dose. The equation used was the 3-parameter logistic fit. No constraints were applied in the calculations. The equation in GraphPad Prism is denoted as Y = Bottom + (Top - Bottom)/(1 + [X/IC50]).

## 3 Results

### 3.1 Novel compounds are selective for mTORC1 over mTORC2

Since mTORC1 hyperactivation has been implicated in certain mTORopathies such as TSC, three mTORC1-selective inhibitors were developed and will be referred to as compounds 425, 276, and 402. mTORC1-selective inhibitory action of these compounds was validated in PC3 cells using an AlphaLISA assays. PC3 cells were selected for the primary assay to identify novel compounds that inhibit mTORC1 selectively based on previously published work that showed PC3 cells are the most sensitive cell line, compared to HEK-293T, HeLa, and H460 cells, for detecting mTORC2 inhibition (Sarbassov et al., 2006). AlphaLISA is a technique used to quantify protein levels that improves upon the traditional ELISA assay by quantifying energy transfer between donor and acceptor beads conjugated to specific antibodies, improving sensitivity and reducing variability (Bielefeld-Sevigny, 2009). Since mTORC1 typically mediates S6K phosphorylation while mTORC2 mediates Akt phosphorylation (pAKT, Ser473) (Hay and Sonenberg, 2004; Sarbassov et al., 2005), AlphaLISA assays specific to p70 S6K and pAKT (pS473) were used to quantify levels of these phospho-proteins, thus quantifying the level of inhibition of mTORC1 and mTORC2. The results of the AlphaLISA against p70 S6K showed similar inhibition of mTORC1 as rapamycin (Figure 1A), with a slight shift toward reduced potency. However, AlphaLISA results against pAKT (pS473) showed a lack of inhibition by the three compounds in the same dose range as rapamycin (Figure 1B). The IC_50_ values for each compound are shown in Table 2. These data show that the three compounds are selective for mTORC1 inhibition in the same dose range that rapamycin inhibits both mTORC1 and mTORC2 in PC3 cells. The apparent activation of AKT signaling caused by rapamycin at very low concentrations and by the selective compounds at intermediate concentrations may be due to the loss of negative feedback control of mTORC1 on mTORC2.

**TABLE 2:** AlphaLISA IC_50_ values for mammalian target of rapamycin complex 1 (mTORC1) and mammalian target of rapamycin complex 2 (mTORC2) targets.

Target	IC_50_ (nM)			
	Rapamycin A	EOAI3973402 ‘402’	EOAI3978276 ‘276’	EOAI3981425 ‘425’
mTORC1 IC_50_ (M)	2.820e-011	3.761e-010	2.191e-010	3.629e-010
mTORC2 IC_50_ (M)	8.440e-009	NC	NC	NC

### 3.2 Novel mTORC1-selective inhibitors prevent hyperexcitability in *TSC2*^–/–^ neurons

**FIGURE 2:**
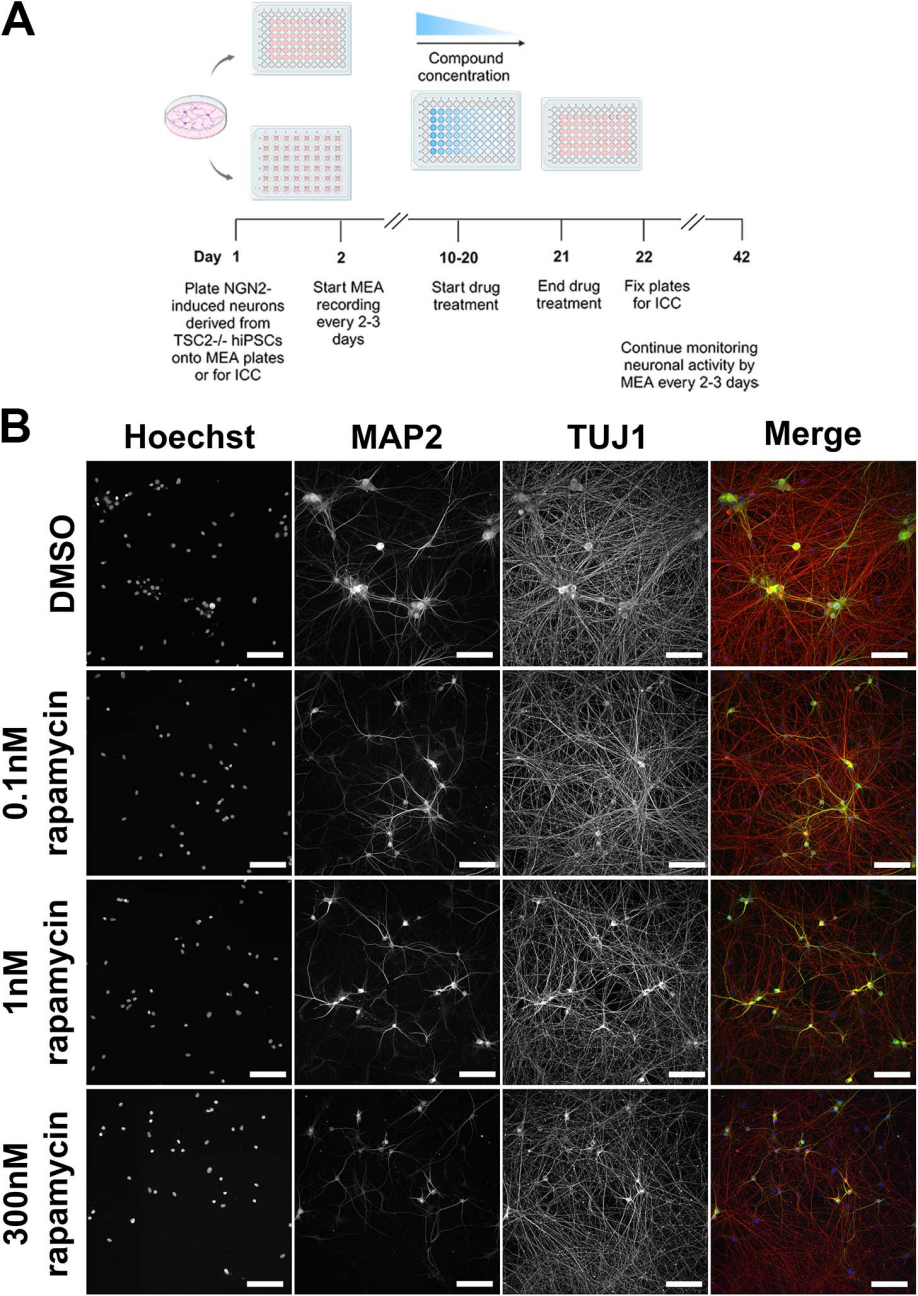
Dose response treatment design and representative images with rapamycin dose-response treatment. **(A)** Schematic for the experiment. *TSC2^–/–^* human induced pluripotent stem cells (iPSCs) were differentiated to neurons by overexpressing NGN2. These neurons were replated along with commercially available astrocytes onto multi-electrode array (MEA) plates to monitor neuronal activity as well as onto 96 well plates for immunocytochemistry (ICC). Treatment with various concentrations of the mTORC1-selective inhibitors or with rapamycin was started around day 10 and continued until day 21. Recordings were performed every 2–3 days starting from day 2 of plating until day 42. Cells were fixed for staining on 1 day after the treatment period ended, on day 22. **(B)** Representative images of *TSC2^–/–^* iPSC-derived neurons fixed on day 22 following 2 weeks of Rapamycin treatment at the doses listed (DMSO control, 0.1 nM Rapamycin, 1 nM Rapamycin, 300 nM Rapamycin) with immunohistochemistry against MAP2, TUJ1, and nuclear stain HOECHST. Scale bar = 100 μm. Schematic in **A** was generated using BioRender.

**FIGURE 3:**
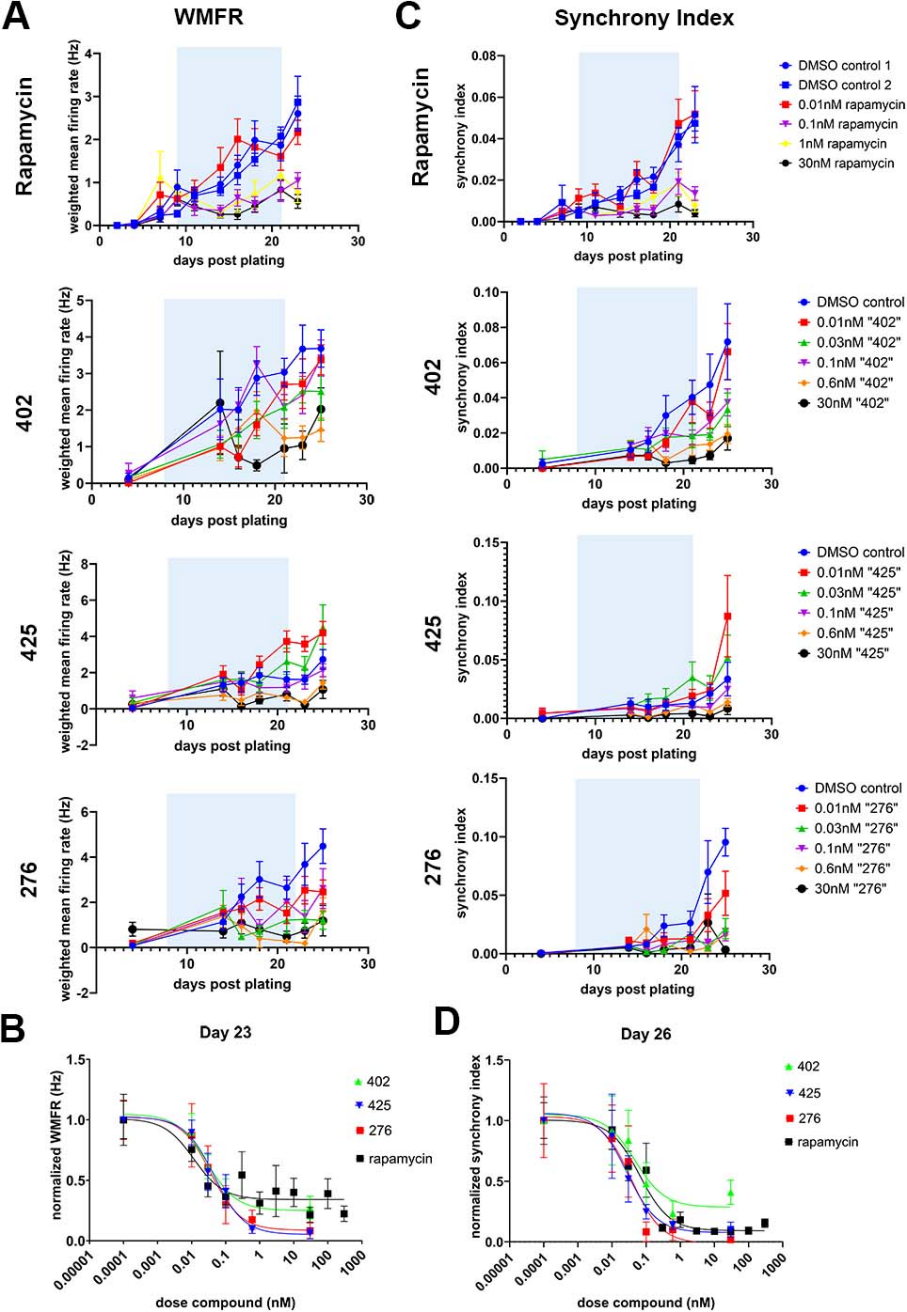
Dose response of novel mammalian target of rapamycin complex 1 (mTORC1)-selective inhibitors on neuronal activity. **(A)** Weighted mean firing rate (WMFR) over time for select doses of mTORC1-selective inhibitors or of rapamycin with the treatment window highlighted in blue. **(B)** Dose response curve to determine the WMFR IC_50_ of neurons on day 23 upon treatment with the mTORC1-selective inhibitors 276, 402, 425 or with rapamycin for 2 weeks until day 21. **(C)** Synchrony index over time for select doses of mTORC1-selective inhibitors or of rapamycin with the treatment window highlighted in blue. **(D)** Synchrony IC_50_ curves on day 26 of the experiment where neurons were treated with mTORC1-selective inhibitors or with rapamycin. Representative data from one of two batches shown here. Data shown as mean ± s.e.m. of 8 technical replicates for each dose at each time point except on day 4 where outliers were excluded due to errors during recording the plate in the first few days post-plating, and 2–8 technical replicates were plotted instead. For **(B,D)**, data was normalized to DMSO-treated controls. Representative curves from one of two batches has been shown; data represented as mean ± s.e.m. of 4–8 technical replicates.

To assess whether these novel mTORC1-selective inhibitors affect disease phenotypes in a relevant cell type, we differentiated *TSC2****^–/–^*** iPSCs to neurons by overexpressing NGN2 (Zhang et al., 2013). NGN2 overexpression has been shown to induce differentiation of iPSCs to excitatory glutamatergic neurons, including expression of markers of both peripheral glutamatergic neurons and cortical glutamatergic neurons (Lin et al., 2021). *TSC2****^–/–^*** iPSC-derived neurons have been shown to exhibit disease-relevant phenotypes such as hyperexcitability and increased soma sizes (Winden et al., 2019). Furthermore, rapamycin treatment for 2 weeks has been shown to rescue these phenotypes by preventing development of neuronal hyperexcitability as well as by preventing changes in soma size (Winden et al., 2019). To assess whether the novel mTORC1-selective inhibitors would rescue these phenotypes, similar to rapamycin, we treated *TSC2****^–/–^*** neurons with varying doses of each of the three novel compounds or with rapamycin (from 0.01 to 30 nM) for 2 weeks and assessed spontaneous neuronal activity by MEA over a period of 42 days with recordings performed every 2–3 days (Figure 2A). Representative images across doses suggested that overt toxicity was not observed from chronic treatment of the compounds at any dose (Figure 2B). Importantly, the weighted mean firing rate (WMFR), indicative of spontaneous neuronal activity, was lowered upon > 0.1 nM rapamycin treatment, consistent with our previous work (Winden et al., 2019) (Figure 3A). Similar to rapamycin, the three novel mTORC1-selective inhibitor compounds also lowered WMFR during the treatment window (Figure 3A) at 0.1 nM or greater doses. The WMFR typically stabilizes a few weeks after plating. Hence, we chose to assess the dose-dependent effects of the mTORC1-selective inhibitors on the WMFR 3 weeks after plating, on day 23, by which time the treatment period had ended, and the compounds had been washed out for a brief period of time. We observed a dose-dependent effect on the WMFR upon treatment with each of the novel compounds similar to that observed upon rapamycin treatment (Figure 3B), suggesting that these compounds rescue neuronal hyperexcitability. The IC_50_ values for each replicate batch on day 23 are shown in Table 3.

**TABLE 3:** Weighted mean firing rate IC_50_ across replicate batches on day 23 of differentiation.

Compound	IC_50_ (nM)			
	Replicate batch #1	Replicate batch #2	Average	Std deviation
Rapamycin	0.015	N/A	–	–
402	0.028	0.030	0.029	0.0016
425	0.027	0.046	0.036	0.014
276	0.037	0.014	0.025	0.017

We observed robust network formation in these cultures by days 21–28 of plating, and chose to assess synchronous firing, an indication of network activity, by measuring the synchrony index. All three compounds reduced the synchrony index relative to DMSO treated controls at doses higher than 0.1 nM, similar to rapamycin (Figure 3C). On day 26, after network formation was observed and the treatment window had ended, once again, we observed a dose dependent effect of the three compounds on the synchrony, similar to rapamycin, although higher doses compound 402 exhibited a lower decrease in synchrony (Figure 3D). The IC_50_ values for each replicate batch on day 26 are shown in Table 4. Taken together, all three mTORC1-selective inhibitors alter neuronal activity as indicated by decreased WMFR and synchrony index and display dose-dependent effects, similar to that observed upon rapamycin treatment.

**TABLE 4:** Synchrony index IC_50_ across replicate batches on day 26 of differentiation.

Compound	IC_50_ (nM)			
	Replicate batch #1	Replicate batch #2	Average	Std deviation
Rapamycin	0.065	N/A	–	–
402	0.048	0.026	0.037	0.015
425	0.0094	0.028	0.019	0.013
276	0.044	0.0094	0.027	0.025

Activity of the neuronal cultures was recorded for several weeks beyond the treatment period to determine if the effects on activity were long-lasting. Interestingly, it appears that the differences across doses remained, but the activity and synchrony continued to increase post-treatment in all conditions (Supplementary Figure 2).

### 3.3 Novel mTORC1-selective inhibitors partially rescue neuronal morphology

**FIGURE 4:**
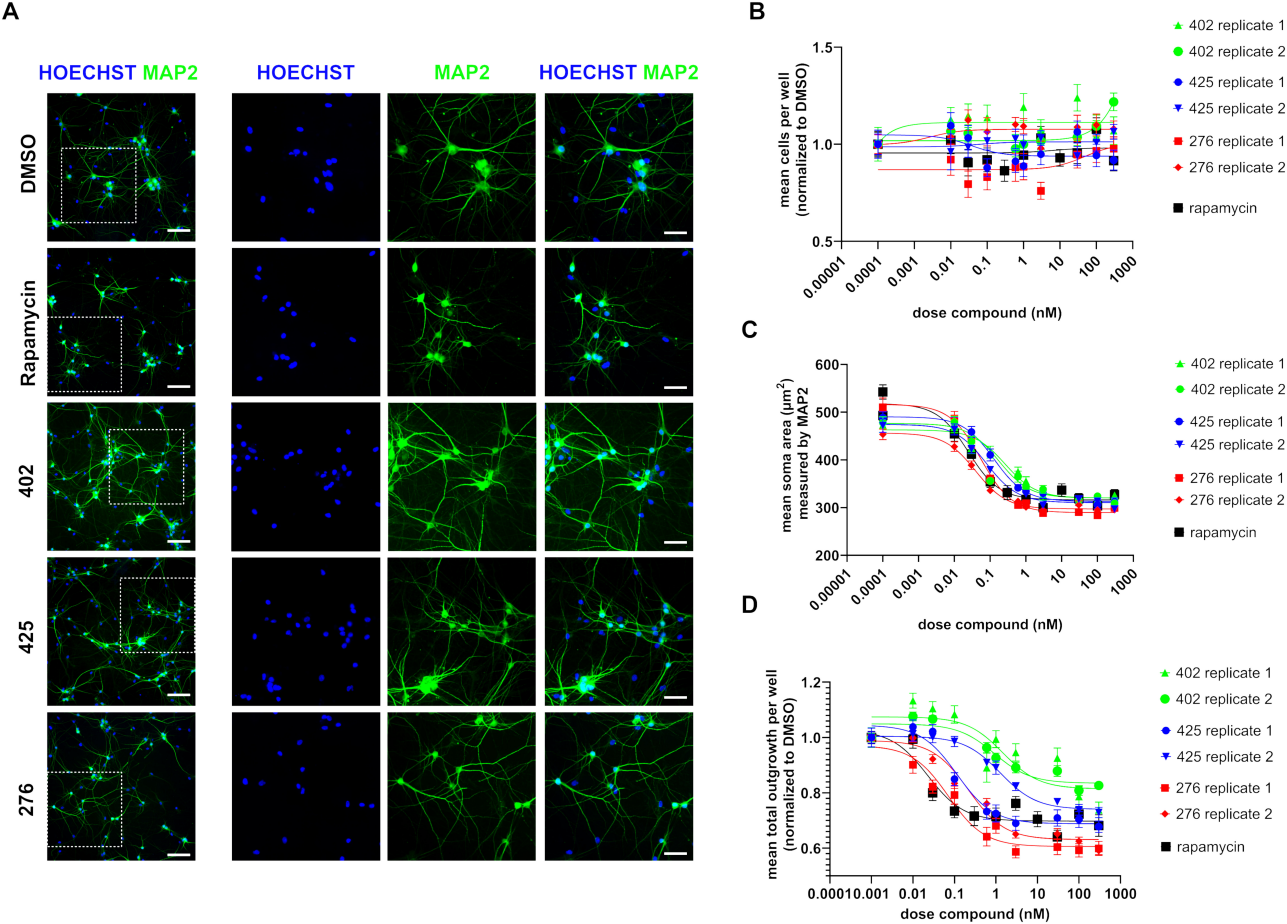
Effect of novel mammalian target of rapamycin complex 1 (mTORC1)-selective inhibitors on cell morphology. **(A)** Representative MAP2 immunofluorescence staining of neurons treated with 300 nM of either rapamycin or of each of the mTORC1-selective inhibitors-compounds 402, 425, 276. Panel on the right is a magnified version of the region highlighted in white on the left. Scale bar = 100 μm for images on the left and 50 μm for the images on the right. **(B)** Average number of cells per well normalized to DMSO-treated controls measured by quantifying nuclear Hoechst staining. **(C)** Dose response of the compounds on cell size measured by quantifying MAP2+ soma area. **(D)** Dose response of compounds on neurite outgrowth as assessed by MAP2 staining normalized to DMSO-treated controls. For **(B–D)**, data from each of the two batches of neurons has been shown separately. Data represented as mean ± s.e.m. of 9–18 technical replicates per batch; this data was collected from two technical replicate wells per batch with 9 fields of view imaged per well and outlier wells with debris were excluded from the analysis.

*TSC2* deficiency is marked by increased soma sizes and neurite lengths in NGN2-derived neurons (Winden et al., 2019). These phenotypes are rescued upon rapamycin treatment. To assess if the novel compounds altered cell morphology, we measured the soma area and the total neurite outgrowth by MAP2 immunostaining (Figure 4A) at the conclusion of the 2 weeks treatment. Automated image analysis was used to quantify soma size and neurite length (Supplementary Figure 3). Importantly, we observed no adverse effects of the compounds/rapamycin treatment on cell viability across various doses (Figure 4B). Next, we observed that similar to rapamycin, all three compounds reduced soma sizes, across two independent batches of treatment (Figure 4C). Total neurite length per well was also decreased by all three compounds, relative to the DMSO treated control wells. However, only compound 276 lowered the total neurite outgrowth similar to rapamycin (Figure 4D), while compound 425 exhibited variability in the neurite outgrowth reduction. Overall, although not all compounds had effects similar to that of rapamycin treatment, all three mTORC1-selective inhibitors altered cellular morphology as indicated by decreased soma sizes and neurite lengths.

## 4 Discussion

Disruption of mTOR signaling due to variants in its repressors such as *TSC1*, *TSC2*, *DEPDC5*, *PTEN* or in its activators such as *RHEB*, and *MTOR* lead to a set of disorders characterized by early onset and often drug-resistant epilepsies called mTORopathies (Lipton and Sahin, 2014; Moloney et al., 2021). While rapalogs have been shown to reduce seizure frequency in a subset of patients with mTORopathies, their effectiveness in improving cognitive functioning is not clear (Krueger et al., 2017; Moloney et al., 2023; Overwater et al., 2019; Srivastava et al., 2022), indicating a need for the development of additional therapeutics targeting mTOR signaling.

Mechanistic target of rapamycin signaling occurs through two major complexes: mTORC1 and mTORC2, each formed in response to diverse cellular stimuli, with different effects on several processes including synaptic functioning and dendritic morphology (McCabe et al., 2020; Switon et al., 2017; Urbanska et al., 2012). Different mTORopathies exhibit distinct patterns of mTOR complex dysregulation. For instance, while mTORC1 hyperactivation has been predominantly implicated in TSC (Costa et al., 2016), PTEN deficiency is thought to cause hyperactivation of mTORC1 as well as mTORC2 (Cullen et al., 2023; D’Amore et al., 2025; Dhaliwal et al., 2024). A careful understanding of the effects of these mTOR complexes on cellular functioning and disease phenotypes would facilitate precise therapeutic targeting for each variant. As such, there is a need to distinguish between mTORC1 and mTORC2 activities and assess functional outcomes in disease-relevant cell types upon treatment with modulators of mTOR signaling. Here, we report the effects of three novel mTORC1-selective inhibitors on disease phenotypes in *TSC2****^–/–^*** human iPSC-derived neurons, including hyperactivity, soma size, and neurite outgrowth.

One caveat with the AlphaLISA experiments used to establish mTORC1-selectivity of the novel compounds is that only one downstream phospho-target of each mTORC1 and mTORC2 were assessed. It is known that rapamycin incompletely inhibits phosphorylation of some mTORC1 substrates, such as ULK1 and 4E-BP (Choo et al., 2008; Kang et al., 2013; Yoon and Roux, 2013). Therefore, future western blot experiments should be completed to determine whether the novel compounds have any activity on targets of mTORC1 that are not inhibited by rapamycin.

Mechanistic target of rapamycin hyperactivation has been suggested to increase soma size in several rodent and human iPSC-based studies with rapamycin rescuing this phenotype (Costa et al., 2016; Karalis et al., 2022; Weston et al., 2014). Consistent with those studies, we found that alterations in cellular morphology were rescued upon mTORC1-selective inhibitor treatment. Since the neuronal activity and synchrony index continued to increase post-treatment in all treatment groups, we hypothesized that the compounds were not causing toxicity to the neurons despite potentially confounding results showing decreased soma size and neurite outgrowth, which we are attributing to changes in mTOR activity. However, to definitively address this question, future studies could utilize live/dead stains to ensure that compound treatment is not causing any neuronal toxicity.

Similar to the image-based endpoints, hyperexcitability and network level synchrony of these cells were significantly reduced in a manner similar to that observed with rapamycin treatment. Overall, mTORC1-selective inhibitors nearly completely rescued morphological and functional deficits introduced by *TSC2* deficiency.

In this work we focused on the cell-autonomous effects of mTOR inhibition in excitatory neurons that were cultured on a feeder layer of WT astrocytes. Since mTORC1 hyperactivation in TSC has been suggested to affect astrocyte proliferation and functioning (Li et al., 2017; Wong and Crino, 2012) and astrocytes play an important role in mediating synaptic functioning, it would be of interest to study the effects of the inhibitors on the hyperexcitability phenotype in co-cultures of *TSC2****^–/–^*** neurons and astrocytes. Moreover, since NGN2 overexpression predominantly generates excitatory neurons, alternative protocols to generate inhibitory neurons (Sun et al., 2016; Yang et al., 2017) can be employed to assess the effects of these compounds on inhibitory neurons or co-cultures of inhibitory and excitatory neurons, since an imbalance in excitation/inhibition has been reported in TSC-deficient rodent models (Bateup et al., 2013).

Overall, our study indicated that, similar to rapamycin, the mTORC1-selective inhibitors exerted protective effects on the *TSC2****^–/–^*** iPSC-derived neurons as they rescued disease phenotypes (neuronal hyperexcitability and abnormal morphology); the effects were very similar in magnitude to rapamycin, as demonstrated by their very similar IC_50_. Future studies should also include the assessment of mTORC2-selective inhibitors to understand which cellular features are capable of modulation from this mechanism. As it is, the assays presented here provide a valuable framework for assessing human neuronal responses to potentially disease-modifying therapeutics and can be used for identification of other potential novel therapeutics for TSC.

## Data Availability

The original contributions presented in this study are included in this article/Supplementary material, further inquiries can be directed to the corresponding author.

## Supplementary material

Supplementary Figure 1 Broad Markush structure of novel mTORC1-selective inhibitors from US patent US11230557B2. The three compounds used in this study differ in the types of substitutions made in the positions R1, R2, and R3. Each compound represents a different type of substitution in the R1-R3 positions to convey selectivity of the compounds to inhibiting mTORC1 versus mTORC2.

Supplementary Figure 2 Effect of novel mTORC1-selective inhibitors on neuronal activity over time. (A) Weighted mean firing rate (WMFR), and (B) synchrony index measured over the course of the entire experiment, including a washout period from day 22 until day 42 for neurons treated with certain doses of rapamycin. The treatment window has been highlighted in blue. Representative data from one of two batches shown here. Data shown as mean s.e.m. of eight technical replicates for each dose at each time point except on day 4 where outliers were excluded due to errors during recording the plate in the first few days post-plating, and 2-8 technical replicates were plotted instead.

Supplementary Figure 3 Soma size and neurite outgrowth quantification. (A) Representative MAP2 immunofluorescence staining with MAP2 positive soma identified using Image Express Micro Confocal’s custom analysis module. Scale bar = 53 m. (B) Representative MAP2 staining with neurites identified using MetaXpress neurite outgrowth analysis module. The region highlighted in white in the image on the left has been magnified in the panel on the right for visualization of neurites. The thick white areas around the cell bodies are excluded from neurite length quantification. Scale bar = 100 m for the image on the left and 50 m for the image panel on the right.

## References

[B1] Afshar Saber W. Sahin M. (2020). Recent advances in human stem cell-based modeling of Tuberous Sclerosis Complex. *Mol Autism* 11:16. 10.1186/s13229-020-0320-2 32075691 PMC7031912

[B2] Balgi A. D. Fonseca B. D. Donohue E. Tsang T. C. Lajoie P. Proud C. G. (2009). Screen for chemical modulators of autophagy reveals novel therapeutic inhibitors of mTORC1 signaling. *PLoS One* 4:e7124. 10.1371/journal.pone.0007124 19771169 PMC2742736

[B3] Bateup H. S. Johnson C. A. Denefrio C. L. Saulnier J. L. Kornacker K. Sabatini B. L. (2013). Excitatory/inhibitory synaptic imbalance leads to hippocampal hyperexcitability in mouse models of tuberous sclerosis. *Neuron* 78 510–522. 10.1016/j.neuron.2013.03.017 23664616 PMC3690324

[B4] Bielefeld-Sevigny M. (2009). AlphaLISA immunoassay platform- the “no-wash” high-throughput alternative to ELISA. *Assay Drug Dev. Technol.* 7 90–92. 10.1089/adt.2009.9996 19382891

[B5] Bissler J. J. Kingswood J. C. Radzikowska E. Zonnenberg B. A. Belousova E. Frost M. D. (2017). Everolimus long-term use in patients with tuberous sclerosis complex: Four-year update of the EXIST-2 study. *PLoS One* 12:e0180939. 10.1371/journal.pone.0180939 28792952 PMC5549893

[B6] Choo A. Y. Yoon S. O. Kim S. G. Roux P. P. Blenis J. (2008). Rapamycin differentially inhibits S6Ks and 4E-BP1 to mediate cell-type-specific repression of mRNA translation. *Proc. Natl. Acad. Sci. U. S. A.* 105 17414–17419. 10.1073/pnas.0809136105 18955708 PMC2582304

[B7] Costa V. Aigner S. Vukcevic M. Sauter E. Behr K. Ebeling M. (2016). mTORC1 inhibition corrects neurodevelopmental and synaptic alterations in a human stem cell model of tuberous sclerosis. *Cell Rep.* 15 86–95. 10.1016/j.celrep.2016.02.090 27052171

[B8] Cullen E. R. Tariq K. Shore A. N. Luikart B. W. Weston M. C. (2023). mTORC2 inhibition improves morphological effects of PTEN Loss, but does not correct synaptic dysfunction or prevent seizures. *J. Neurosci.* 43 827–845. 10.1523/JNEUROSCI.1354-22.2022 36526374 PMC9899090

[B9] D’Amore A. Sundberg M. Lin R. Lubbers E. T. Winden K. D. Yu L. (2025). Phenotypic rescue via mTOR inhibition in neuron-specific Pten knockout mice reveals AKT and mTORC1-site specific changes. *Mol. Psychiatry* 30 3077–3089. 10.1038/s41380-025-02916-2 39953287

[B10] Dhaliwal N. K. Weng O. Y. Dong X. Bhattacharya A. Ahmed M. Nishimura H. (2024). Synergistic hyperactivation of both mTORC1 and mTORC2 underlies the neural abnormalities of PTEN-deficient human neurons and cortical organoids. *Cell Rep.* 43:114173. 10.1016/j.celrep.2024.114173 38700984

[B11] Di Nardo A. Lenoel I. Winden K. D. Ruhmkorf A. Modi M. E. Barrett L. (2020). Phenotypic screen with TSC-deficient neurons reveals heat-shock machinery as a druggable pathway for mtORC1 and reduced cilia. *Cell Rep.* 31:107780. 10.1016/j.celrep.2020.107780 32579942 PMC7381997

[B12] Ebrahimi-Fakhari D. Saffari A. Wahlster L. DiNardo A. Turner D. Lewis T. L. Jr. (2016). Impaired mitochondrial dynamics and mitophagy in neuronal models of tuberous sclerosis complex. *Cell Rep.* 17:2162. 10.1016/j.celrep.2016.10.051 27851977

[B13] Hay N. Sonenberg N. (2004). Upstream and downstream of mTOR. *Genes Dev.* 18 1926–1945. 10.1101/gad.1212704 15314020

[B14] Huang J. Dibble C. C. Matsuzaki M. Manning B. D. (2008). The TSC1-TSC2 complex is required for proper activation of mTOR complex 2. *Mol. Cell Biol.* 28 4104–4115. 10.1128/MCB.00289-08 18411301 PMC2423120

[B15] Huang J. Manning B. D. (2008). The TSC1-TSC2 complex: A molecular switchboard controlling cell growth. *Biochem. J.* 412 179–190. 10.1042/BJ20080281 18466115 PMC2735030

[B16] Huang J. Manning B. D. (2009). A complex interplay between Akt, TSC2 and the two mTOR complexes. *Biochem. Soc. Trans.* 37(Pt 1), 217–222. 10.1042/BST0370217 19143635 PMC2778026

[B17] Kang S. A. O’Neill D. J. Machl A. W. Lumpkin C. J. Galda S. N. Sengupta S. (2019). Discovery of small-molecule selective mTORC1 inhibitors via direct inhibition of glucose transporters. *Cell Chem. Biol.* 26:e1213. 10.1016/j.chembiol.2019.05.009 31231029

[B18] Kang S. A. Pacold M. E. Cervantes C. L. Lim D. Lou H. J. Ottina K. (2013). mTORC1 phosphorylation sites encode their sensitivity to starvation and rapamycin. *Science* 341:1236566. 10.1126/science.1236566 23888043 PMC3771538

[B19] Karalis V. Caval-Holme F. Bateup H. S. (2022). Raptor downregulation rescues neuronal phenotypes in mouse models of Tuberous Sclerosis Complex. *Nat. Commun.* 13:4665. 10.1038/s41467-022-31961-6 35945201 PMC9363483

[B20] Krueger D. A. Sadhwani A. Byars A. W. de Vries P. J. Franz D. N. Whittemore V. H. (2017). Everolimus for treatment of tuberous sclerosis complex-associated neuropsychiatric disorders. *Ann. Clin. Transl. Neurol.* 4 877–887. 10.1002/acn3.494 29296616 PMC5740257

[B21] Lamming D. W. (2016). Inhibition of the mechanistic target of Rapamycin (mTOR)-Rapamycin and beyond. *Cold Spring Harb. Perspect. Med.* 6:a025924. 10.1101/cshperspect.a025924 27048303 PMC4852795

[B22] Lamming D. W. Ye L. Katajisto P. Goncalves M. D. Saitoh M. Stevens D. M. (2012). Rapamycin-induced insulin resistance is mediated by mTORC2 loss and uncoupled from longevity. *Science* 335 1638–1643. 10.1126/science.1215135 22461615 PMC3324089

[B23] Li Y. Cao J. Chen M. Li J. Sun Y. Zhang Y. (2017). Abnormal neural progenitor cells differentiated from induced pluripotent stem cells partially mimicked development of TSC2 neurological abnormalities. *Stem Cell Rep.* 8 883–893. 10.1016/j.stemcr.2017.02.020 28344003 PMC5390135

[B24] Lin H. C. He Z. Ebert S. Schornig M. Santel M. Nikolova M. T. (2021). NGN2 induces diverse neuron types from human pluripotency. *Stem Cell Rep.* 16 2118–2127. 10.1016/j.stemcr.2021.07.006 34358451 PMC8452516

[B25] Lipton J. O. Sahin M. (2014). The neurology of mTOR. *Neuron* 84 275–291. 10.1016/j.neuron.2014.09.034 25374355 PMC4223653

[B26] Mahoney S. J. Narayan S. Molz L. Berstler L. A. Kang S. A. Vlasuk G. P. (2018). A small molecule inhibitor of Rheb selectively targets mTORC1 signaling. *Nat. Commun.* 9:548. 10.1038/s41467-018-03035-z 29416044 PMC5803267

[B27] McCabe M. P. Cullen E. R. Barrows C. M. Shore A. N. Tooke K. I. Laprade K. A. (2020). Genetic inactivation of mTORC1 or mTORC2 in neurons reveals distinct functions in glutamatergic synaptic transmission. *Elife* 9:e51440. 10.7554/eLife.51440 32125271 PMC7080408

[B28] Moloney P. B. Cavalleri G. L. Delanty N. (2021). Epilepsy in the mTORopathies: Opportunities for precision medicine. *Brain Commun.* 3:fcab222. 10.1093/braincomms/fcab222 34632383 PMC8495134

[B29] Moloney P. B. Kearney H. Benson K. A. Costello D. J. Cavalleri G. L. Gorman K. M. (2023). Everolimus precision therapy for the GATOR1-related epilepsies: A case series. *Eur. J. Neurol.* 30 3341–3346. 10.1111/ene.15975 37422919

[B30] Overwater I. E. Rietman A. B. van Eeghen A. M. de Wit M. C. Y. (2019). Everolimus for the treatment of refractory seizures associated with tuberous sclerosis complex (TSC): Current perspectives. *Ther. Clin. Risk Manag.* 15 951–955. 10.2147/TCRM.S145630 31440057 PMC6666377

[B31] Parenti I. Rabaneda L. G. Schoen H. Novarino G. (2020). Neurodevelopmental disorders: From genetics to functional pathways. *Trends Neurosci.* 43 608–621. 10.1016/j.tins.2020.05.004 32507511

[B32] Reijnders M. R. F. Kousi M. van Woerden G. M. Klein M. Bralten J. Mancini G. M. S. (2017). Variation in a range of mTOR-related genes associates with intracranial volume and intellectual disability. *Nat. Commun.* 8:1052. 10.1038/s41467-017-00933-6 29051493 PMC5648772

[B33] Rosset C. Netto C. B. O. Ashton-Prolla P. (2017). TSC1 and TSC2 gene mutations and their implications for treatment in Tuberous Sclerosis Complex: A review. *Genet. Mol. Biol.* 40 69–79. 10.1590/1678-4685-GMB-2015-0321 28222202 PMC5409767

[B34] Sarbassov D. D. Ali S. M. Sengupta S. Sheen J. H. Hsu P. P. Bagley A. F. (2006). Prolonged rapamycin treatment inhibits mTORC2 assembly and Akt/PKB. *Mol. Cell* 22 159–168. 10.1016/j.molcel.2006.03.029 16603397

[B35] Sarbassov D. D. Guertin D. A. Ali S. M. Sabatini D. M. (2005). Phosphorylation and regulation of Akt/PKB by the rictor-mTOR complex. *Science* 307 1098–1101. 10.1126/science.1106148 15718470

[B36] Schreiber K. H. Arriola Apelo S. I. Yu D. Brinkman J. A. Velarde M. C. Syed F. A. (2019). A novel rapamycin analog is highly selective for mTORC1 in vivo. *Nat. Commun.* 10:3194. 10.1038/s41467-019-11174-0 31324799 PMC6642166

[B37] Srivastava S. Jo B. Zhang B. Frazier T. Gallagher A. S. Peck F. (2022). A randomized controlled trial of everolimus for neurocognitive symptoms in PTEN hamartoma tumor syndrome. *Hum. Mol. Genet.* 31 3393–3404. 10.1093/hmg/ddac111 35594551 PMC9558845

[B38] Sun Y. Pasca S. P. Portmann T. Goold C. Worringer K. A. Guan W. (2016). A deleterious Nav1.1 mutation selectively impairs telencephalic inhibitory neurons derived from Dravet Syndrome patients. *Elife* 5:e13073. 10.7554/eLife.13073 27458797 PMC4961470

[B39] Sundberg M. Tochitsky I. Buchholz D. E. Winden K. Kujala V. Kapur K. (2018). Purkinje cells derived from TSC patients display hypoexcitability and synaptic deficits associated with reduced FMRP levels and reversed by rapamycin. *Mol. Psychiatry* 23 2167–2183. 10.1038/s41380-018-0018-4 29449635 PMC6093816

[B40] Switon K. Kotulska K. Janusz-Kaminska A. Zmorzynska J. Jaworski J. (2017). Molecular neurobiology of mTOR. *Neuroscience* 341 112–153. 10.1016/j.neuroscience.2016.11.017 27889578

[B41] Trelinska J. Dachowska I. Kotulska K. Fendler W. Jozwiak S. Mlynarski W. (2015). Complications of mammalian target of rapamycin inhibitor anticancer treatment among patients with tuberous sclerosis complex are common and occasionally life-threatening. *Anticancer Drugs* 26 437–442. 10.1097/CAD.0000000000000207 25719621

[B42] Urbanska M. Gozdz A. Swiech L. J. Jaworski J. (2012). Mammalian target of rapamycin complex 1 (mTORC1) and 2 (mTORC2) control the dendritic arbor morphology of hippocampal neurons. *J. Biol. Chem.* 287 30240–30256. 10.1074/jbc.M112.374405 22810227 PMC3436277

[B43] Vasic V. Jones M. S. O. Haslinger D. Knaus L. S. Schmeisser M. J. Novarino G. (2021). Translating the Role of mTOR- and RAS-associated signalopathies in autism spectrum disorder: Models, mechanisms and treatment. *Genes* 12:1746. 10.3390/genes12111746 34828352 PMC8624393

[B44] Weston M. C. Chen H. Swann J. W. (2014). Loss of mTOR repressors Tsc1 or Pten has divergent effects on excitatory and inhibitory synaptic transmission in single hippocampal neuron cultures. *Front. Mol. Neurosci.* 7:1. 10.3389/fnmol.2014.00001 24574959 PMC3922082

[B45] Winden K. D. Ebrahimi-Fakhari D. Sahin M. (2018). Abnormal mTOR activation in autism. *Annu. Rev. Neurosci.* 41 1–23. 10.1146/annurev-neuro-080317-061747 29490194

[B46] Winden K. D. Pham T. T. Teaney N. A. Ruiz J. Chen R. Chen C. (2023). Increased degradation of FMRP contributes to neuronal hyperexcitability in tuberous sclerosis complex. *Cell Rep.* 42:112838. 10.1016/j.celrep.2023.112838 37494191 PMC10529098

[B47] Winden K. D. Sundberg M. Yang C. Wafa S. M. A. Dwyer S. Chen P. F. (2019). Biallelic Mutations in TSC2 lead to abnormalities associated with cortical tubers in human iPSC-derived neurons. *J. Neurosci.* 39 9294–9305. 10.1523/JNEUROSCI.0642-19.2019 31591157 PMC6867816

[B48] Wong M. Crino P. B. (2012). Tuberous sclerosis and epilepsy: Role of astrocytes. *Glia* 60 1244–1250. 10.1002/glia.22326 22438024

[B49] Yang N. Chanda S. Marro S. Ng Y. H. Janas J. A. Haag D. (2017). Generation of pure GABAergic neurons by transcription factor programming. *Nat. Methods* 14 621–628. 10.1038/nmeth.4291 28504679 PMC5567689

[B50] Yang Z. Teaney N. A. Buttermore E. D. Sahin M. Afshar-Saber W. (2024). Harnessing the potential of human induced pluripotent stem cells, functional assays and machine learning for neurodevelopmental disorders. *Front. Neurosci.* 18:1524577. 10.3389/fnins.2024.1524577 39844857 PMC11750789

[B51] Yoon S. O. Roux P. P. (2013). Rapamycin resistance: mTORC1 substrates hold some of the answers. *Curr. Biol.* 23 R880–R883. 10.1016/j.cub.2013.08.030 24112984

[B52] Zhang Y. Pak C. Han Y. Ahlenius H. Zhang Z. Chanda S. (2013). Rapid single-step induction of functional neurons from human pluripotent stem cells. *Neuron* 78 785–798. 10.1016/j.neuron.2013.05.029 23764284 PMC3751803

