# Supplementary figures and images for: mTORC1-selective inhibitors rescue cellular phenotypes in TSC iPSC-derived neurons
Source: Front Neurosci. 2025 Jul 28;19:1595880. doi: 10.3389/fnins.2025.1595880 (PMC12337281; doi:10.3389/fnins.2025.1595880)

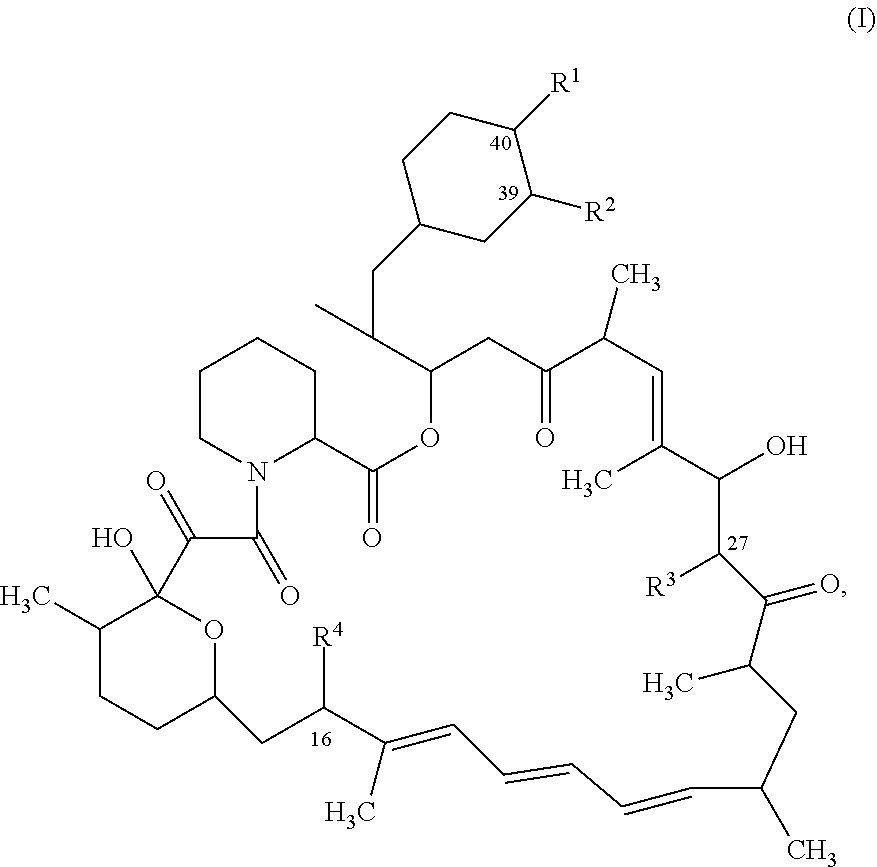

Supplement: Supplementary Figure 1 — Broad Markush structure of novel mTORC1-selective inhibitors from US patent US11230557B2. The three compounds used in this study differ in the types of substitutions made in the positions R1, R2, and R3. Each compound represents a different type of substitution in the R1-R3 positions to convey selectivity of the compounds to inhibiting mTORC1 versus mTORC2. [file Image_1.tif]

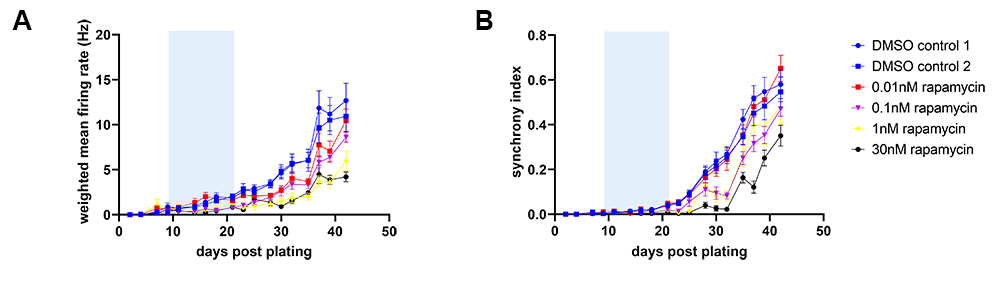

Supplement: Supplementary Figure 2 — Effect of novel mTORC1-selective inhibitors on neuronal activity over time. (A) Weighted mean firing rate (WMFR), and (B) synchrony index measured over the course of the entire experiment, including a washout period from day 22 until day 42 for neurons treated with certain doses of rapamycin. The treatment window has been highlighted in blue. Representative data from one of two batches shown here. Data shown as mean s.e.m. of eight technical replicates for each dose at each time point except on day 4 where outliers were excluded due to errors during recording the plate in the first few days post-plating, and 2-8 technical replicates were plotted instead. [file Image_2.tif]

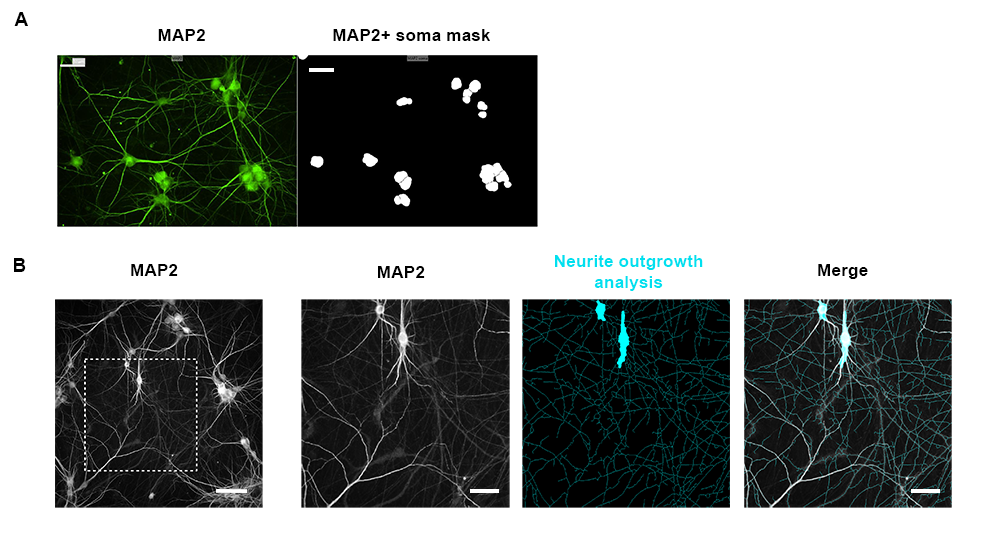

Supplement: Supplementary Figure 3 — Soma size and neurite outgrowth quantification. (A) Representative MAP2 immunofluorescence staining with MAP2 positive soma identified using Image Express Micro Confocal’s custom analysis module. Scale bar = 53 m. (B) Representative MAP2 staining with neurites identified using MetaXpress neurite outgrowth analysis module. The region highlighted in white in the image on the left has been magnified in the panel on the right for visualization of neurites. The thick white areas around the cell bodies are excluded from neurite length quantification. Scale bar = 100 m for the image on the left and 50 m for the image panel on the right. [file Image_3.tif]
